# Validity and Reliability of Vis-Screen Application: A Smartphone-Based Distance Vision Testing for Visual Impairment and Blindness Vision Screening

**DOI:** 10.3390/medicina59050912

**Published:** 2023-05-10

**Authors:** Siti Nor Aishah Abdul Rahman, Nyi Nyi Naing, Abdul Mutalib Othman, Zarina Mohamad, Tg Mohd Masrul Ibrahim, Syaratul Emma Hashim, Atif Amin Baig, Ling Shing Wong, Hemaniswarri Dewi Dewadas, Siddharthan Selvaraj

**Affiliations:** 1 Faculty of Medicine, Medical Campus, Universiti Sultan Zainal Abidin, Jalan Sultan Mahmud, Kuala Terengganu 20400, Terengganu, Malaysia; aishah_kudo@yahoo.com.my (S.N.A.A.R.); othmanabdulmutalib@yahoo.com (A.M.O.); emmahashim@unisza.edu.my (S.E.H.); 2 Faculty of Informatics and Computing, Besut Campus, Universiti Sultan Zainal Abidin, Besut 22200, Terengganu, Malaysia; zarina@unisza.edu.my; 3 Infostructure & Network Management Center, Gong Badak Campus, Universiti Sultan Zainal Abidin, Kuala Nerus 21300, Terengganu, Malaysia; masrul@unisza.edu.my; 4 University Institute of Public Health, Faculty of Allied Health Sciences, The University of Lahore, Lahore 54590, Pakistan; atifamin@unisza.edu.my; 5 Faculty of Health and Life Sciences, INTI International University, Nilai 71800, Negeri Sembilan, Malaysia; 6 Centre for Biomedical and Nutrition Research, Universiti Tunku Abdul Rahman, Jalan Universiti, Banda Barat, Kampar 31900, Perak, Malaysia; 7 Department of Business and Public Administration, Faculty of Business and Finance, Universiti Tunku Abdul Rahman, Jalan Universiti, Banda Barat, Kampar 31900, Perak, Malaysia; 8 Faculty of Dentistry, AIMST University, Bedong 08100, Kedah, Malaysia; sidzcristiano@gmail.com

**Keywords:** validity, reliability, vis screen, blindness vision screening

## Abstract

*Background and Objectives*: The health-related mobile applications (app) might assist in promoting inclusive health and tele-treatment, especially for the less severe diseases. In this paper, a study had been done to determine the app’s reliability in terms of raters and the app’s agreement with the Snellen chart. *Materials and Methods*: A cross-sectional sectional study was conducted between November 2019 and September 2020. Participants were selected via purposive sampling from selected communities in Terengganu state. All participants underwent vision testing with the Vis-Screen app and Snellen chart for validity and reliability accordingly. *Results*: A total of 408 participants were involved, with a mean age of 29.3. The sensitivity of the presenting vision of the right eye (PVR) ranged from 55.6% to 88.4%, with specificity between 94.7% to 99.3%, while the positive and negative predictive values were between 57.9% and 81.7% and 96.8% and 99.0%, respectively. The positive likelihood ratios ranged between 16.73 and 73.89, whereas the negative likelihood ratios ranged from 0.12 to 0.45. The area under the receiver operating characteristic curve (AUC) for all cut-off points ranged between 0.93 and 0.97, and the optimum cut-off point was at 6/12. The kappa values for intra-rater and inter-rater were 0.85 and 0.75, respectively, while the app’s reliability with the Snellen chart was 0.61. *Conclusions*: Vis-Screen was concluded to be valid and reliable for use as a screening tool for detecting individuals with visual impairment and blindness in the community. A valid and reliable portable vision screener like Vis-Screen will help expand the eye care feasibility while providing similar accuracy as the conventional charts in clinical practices.

## 1. Introduction

The essentiality of taking care of one’s eyesight is not particularly highlighted at a certain age but it is consistently performed throughout life. All over the world, vision screening is one of the provisions taken by the authorities as a part of the health care services. Although vision screening does not replace comprehensive assessments, it may help detect vision problems earlier and save one’s vision, with early referral and appropriate treatment [1,2]. Even though some visual impairments might not reverse eyesight back to normal after the appropriate treatment, an early referral from vision screening may aid in improving one’s quality of life. 

The evolution of visual acuity screening in the modernized form of electronic charts and readily downloaded mobile apps for smart devices is continually expanding. The escalating numbers of these apps in the virtual market portray the currently demanding need for a portable vision screener to increase eye care feasibility, particularly in remote and low-resource areas [3]. Not only that, but the availability of these apps also creates the opportunity for telehealth engagement, especially during the recent pandemic [4]. 

Various distance vision testing apps have utilized different forms of charts in their algorithm for screening assessment to mimic the frequent routine practices. These include the most popular Tumbling E, followed by Early Treatment Diabetic Retinopathy Study (ETDRS), and Snellen charts [5]. Even though these existing apps could provide another option for vision screening, most of them need to be appropriately validated, thus raising the biggest concern toward their safety and accuracy [3,6]. Regardless of the platform, a validated app is essential to ensure that the number of false positives and negatives are minimal so that both misleading results and the burden of unnecessary referrals to health providers can be reduced [7,8].

The primary objective of this study was to determine the validity of the Vis-Screen against the gold standard, the Snellen chart, which is commonly used in clinical practices. This was followed by determining the app’s reliability regarding the raters and its agreement with the Snellen chart. We hypothesize Vis-Screen to be an acceptable psychometric property; hence, Vis-Screen is a valid and reliable vision screening tool that is comparable with the Snellen chart for detecting visual impairment and blindness in community vision screening.

## 2. Materials and Methods

### 2.1. Study Design and Participants

This cross-sectional study from November 2019 to September 2020 was conducted as the outreach of eye screening programs across nine local communities from five different districts in Terengganu. The districts involved were Kuala Terengganu, Kuala Nerus, Marang, Hulu Terengganu, and Besut. Any interested individual who voluntarily attended the screening program who was willing to participate in the study and fulfilled the inclusion criteria was selected as a sample. The inclusion criteria were individuals who were aged 4 years and older, physically fit, and able to communicate with reliable mental status. The participant was considered as fulfilling the ‘reliable status’ criteria if he or she could understand the given instructions and demonstration before the beginning of the test. Individuals or minors without their rightful guardian to provide consent and in need of emergency or exceptional care were excluded from the study. No screening was performed to obtain information about any previous diagnosis of the participants. The recruitment was conducted by selecting all interested participants that willingly wanted to participate in the study and fulfilled all the inclusion criteria, without knowing their previous ocular and medical history. 

### 2.2. Sampling Process

All participants were selected by purposive sampling. The sampling process was initiated by selecting the population of individuals who lived in Terengganu state and were aged 4 and above. Next, the sampling frame was performed by selecting the participants from various sites across all districts in the state. To vary the age groups and mimic the individuals that lived in the actual communities, they were deliberately selected from different sites, which involved local villages, higher institutions, and schools. The identified locations were scheduled for visits to conduct the eye screening program. Once the relevant authorities granted permission, the representative of the communities made an announcement about the details of the program.

### 2.3. Application Description and Test Algorithm

Vis-Screen was developed by researchers and eye experts from the University Sultan Zainal Abidin (UniSZA) in June 2018 and designed for smartphone- and tablet-based users as a portable vision screener for community vision screening. The app’s first version was uploaded to the Google Play Store in March 2019, and it was validated by a pilot study afterward [9]. Unlike the other smartphone-based apps, the novelty of pinhole testing was introduced in the Vis-Screen test’s algorithm. All vision tests were carried out based on the 11th International Classification of Disease (ICD) categories of distance visual impairment (VI) and blindness, as endorsed by the World Health Organization (WHO), to familiarize general users and practitioners on the terms used by the standard classification [10]. This app received copyright registration from the Intellectual Property Corporation of Malaysia (MyIPO) in July 2019, and the reference number was CRLY00014390. The Ministry of Education Prototype Research Grant Scheme financially funded the app.

The vision test was conducted by randomly portraying a single letter E one at a time, and the participant needed to point out the letter’s direction as either up, down, left, or right. Then, the examiner would swipe the screen as mentioned accordingly. Five trials were given for each vision level to mimic the usual Snellen vision testing in clinical practices and to reduce the chance of guessing. In the algorithm, Step 1 to Step 4 required testing distances of 1.5 and 3 m to compensate for the smaller size of smartphones and tablets. Steps 1 and 2 referred to the presenting and corrected vision of the right eye, denoted as presenting vision of the right eye (PVR) and corrected vision of the right eye (CVR). Similarly, Steps 3 and 4 were for the left eye, denoted as presenting vison of the left eye (PVL) and corrected vision of the left eye (CVL). The term ‘presenting vision’ refers to the current vision level of the participant, whether unaided or aided with any optical correction, while the term ‘corrected vision’ refers to the condition in which the participant failed at any level, either at Step 1 or Step 3, and was asked to place the pinhole occluder (Step 2 or Step 4). No time limitation was given for the participants to give their responses. However, they were encouraged to guess the direction of the presented E rather than giving a random answer. All results were presented in Snellen fractional form for easier understanding and standardization with what is commonly reported in clinical practices and the literature. 

### 2.4. Visual Acuity Testing

**Figure 1:**
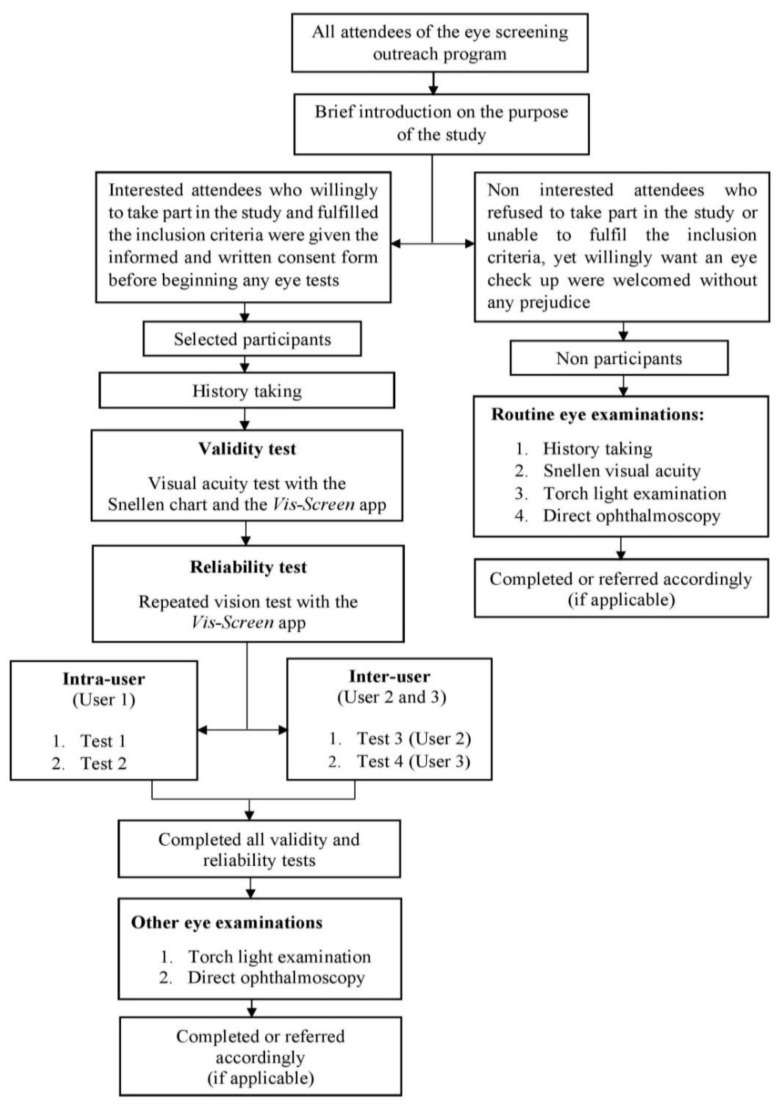
The flowchart of the study protocol.

Every participant underwent two vision tests, with the Vis-Screen app and the Snellen chart (Figure 1). An optometrist was assigned for the Snellen test, while the examiner for the app was either an ophthalmologist, medical officer, paramedic, or medical student. Before the screening, the examiners involved in the app’s test were told to self-download the app and install it on their smart devices. All examiners were encouraged to set the screen brightness of their smartphones or tablets at nearly maximum before beginning the vision test, irrespective of any screen protectors used. They were also told to constantly hold the devices perpendicularly to the participant’s eye level to avoid jeopardizing the participants’ viewing angle.

Each participant was tested unilaterally for the Snellen test, while the non-tested eye was covered with an opaque occluder. Participants with their current corrections were told to keep wearing their optical correction along the measurement taken. A single portable electronic smart chart (M&S Technologies, Inc., 5715 W, Howard Street, Niles, IL 60714, USA) was used to standardize the routine procedure at 6 m testing distance. The optotypes were displayed as a single line that consisted of five different Sloan letters for each visual level. Each participant was asked to continually read the optotypes from the biggest at 6/60 level to the smallest that could be seen. All eye assessments were performed indoors with controlled lighting to avoid any unnecessary glares during the tests. 

For validity, two vision tests were performed for each participant: Vis-Screen and Snellen. No specific randomization was applied for the tests’ order, and the Snellen chart was used as the gold standard throughout this study. Both presenting and corrected vision attained from the app were compared with the distance visual acuity from the Snellen chart, and they were reclassified accordingly (Table 1). All results for visual acuity assessments were retained as Snellen fractions and were not converted into any logarithmic form to avoid misinterpretation of the results.

The reliability test was performed once by repeatedly conducting the same app’s test as intra-rater and inter-rater. For intra-rater, a single examiner repeated the test twice for each participant, and a brief break was given in between the two tests. Meanwhile, the inter-rater part was performed by repeating the vision test with three different examiners. No test order randomization was applied for both intra-rater and inter-rater. All eye assessments were conducted on the same day of the screening program. Routine eye examinations were performed on all attendees without any prejudice. A stopwatch app from the examiner’s smartphone was used to measure the testing time for the app, starting from Step 1 until all steps were completed and results were obtained. 

### 2.5. Sample Size and Statistical Analysis

This study’s expected sensitivity and specificity were 85% and 95%, based on the previous literature [11,12,13]. The disease prevalence was 0.16, calculated according to the WHO ‘World Report on Vision’ in 2019, representing the general visual impairment concerning all age groups [14]. Considering the socio-demographic background of the participants, the anticipated drop-out rate was 20%, with a confidence interval and precision of 95% and 10%, respectively. A web-based sample size calculator was used to calculate the sample size required for the study according to all the parameters given [15]. The sample size required was 384, and all the participants for validity were also selected unanimously for the reliability part. 

The components involved in the validity analysis were sensitivity, specificity, positive and negative predictive values, accuracy, and receiver operating characteristic (ROC) curve analyses. All components from the validity parts aside from the ROC curve were statistically analyzed, as is commonly performed in other diagnostic testing studies [16]. The definitions, cut-off points, and diagnostic terms used throughout the study are listed in Table 1. The reliability analyses were divided into raters (intra-rater and inter-rater) and the app’s reliability against the Snellen chart. Kappa statistics were used for all reliability analyses: Cohen’s kappa was used for the app’s reliability and intra-user, while Fleiss kappa was used for the inter-user [17,18]. The interpretation of kappa values was conducted according to the guidelines proposed by Landis and Koch [19]. Only vision levels attained from the right eyes were analyzed to synchronize with the other literature and avoid including dependent data. A complete dataset, i.e., sex, age, completed vision test with both the app and the Snellen chart, and test duration was required. The final analysis discarded incomplete or indeterminate data, such as incomplete vision tests. Missing values for either age or test duration with the remaining completed tests were identified by the coding -99 in the data entry. All data analyses were performed using Stata statistical software version 16 for Windows 10 (StataCorp, 2019. *Stata Statistical Software: Release 16.* College Station, TX, USA: StataCorp LLC).

## 3. Results

A total of 408 participants were involved in this study; 186 (45.6%) were males, and the other 222 (54.4%) were females, with a mean (SD) age of 29.3 (22.6) years. The youngest participant was 4, while the oldest was 91 years old. Out of the 408 eyes tested with the Snellen chart for presenting vision, 322 eyes had normal vision, 24 eyes had mild VI, 45 eyes had moderate VI, 8 eyes had severe VI, and 9 eyes were blind. The highest sensitivity of the Vis-Screen was at the 6/12 cut-off point for both PVR and CVR, with the sensitivities of 88.4% and 85.4%, respectively. However, the sensitivities of PVR and CVR gradually decreased for the other cut-off points, which ranged between 82.0% and 55.6% for PVR, while CVR ranged between 60.0% and 73.9%. In contrast, notably high specificities were obtained for all PVR and CVR cut-off points. The highest specificity for both PVR and CVR was at the 3/60 cut-off point, with 99.3% for PVR and 99.0% for CVR. 

The highest PPV for PVR and CVR was also at the 6/12 cut-off point, averaging 81.7% and 68.6%, respectively. At the subsequent cut-off points, the PPV of both PVR and CVR generally declined, which ranged from 57.9% to 76.9% for PVR, and 33.3% to 58.6% for CVR. Meanwhile, the NPV values for PVR and CVR were relatively high at all the cut-points. The highest NPV for both PVR and CVR was at the 3/60 cut-off point with a value of 99.0% for PVR and 99.8% for CVR. Overall, the app’s accuracy at all the cut-off points was generally high, with the highest values for both PVR and CVR at the 3/60 cut-off point (Table 2).

The ROC curve analyses were added with the pre-determined cut-off points. However, the primary purpose of the ROC curve analyses was only to observe the optimal cut-off point in discriminating the visually impaired from the normal ones based on our study population. The area under the ROC curve (AUC) values of both the PVR and CVR showed relatively excellent scores for all cut-off points, with a score more than 0.90. The highest positive likelihood ratio for the PVR and CVR was at the 3/60 cut-off point, with the values of 73.89 and 67.50, respectively. Meanwhile, the lowest negative likelihood ratio for both the PVR and CVR was at the 6/12 cut-off point, with the values of 0.12 for PVR and 0.15 for CVR (Table 3). In a nutshell, Vis-Screen was good at discriminating the visually impaired participants, regardless of any cut-off point. The best cut-off for the app was selected at 6/12, based on the trade-off between sensitivity and specificity values aside from other ROC analyses attained. 

For the raters’ reliability findings, the intra-user of the PVR showed an almost perfect score with a kappa value of 0.85, while the substantial agreement for the CVR had a value of 0.79. However, the kappa values for the inter-rater were slightly lower for both PVR and CVR compared with the intra-user. The kappa value for PVR was 0.72, while for CVR it was 0.67, and both values were categorized as having a substantial agreement. On the other hand, an acceptable agreement was attained for the reliability between the app and Snellen chart, with the kappa values of 0.61 and 0.52 for PVR and CVR, respectively. On the other hand, the mean (SD) time taken to complete all Vis-Screen tests was 70.5 (33.3) seconds, with the shortest being 20 s and the longest being 305 s (Table 4). 

## 4. Discussion

The main priority for any new screening tool is accuracy and validity. Despite the abundance of eye testing apps available in the virtual market, the scarcity of validated apps, mainly for visual acuity screening, added another challenge [6]. For this study, the Vis-Screen app had the highest sensitivity of 88% for PVR at the 6/12 cut-off point. Even though the sensitivities were declined for other cut-off points, the overall moderate to high sensitivities obtained were still comparable with other visual acuity apps that utilized a similar approach as ours. For such, the sensitivities of Peek Acuity were between 48% and 78% among school children at the same 6/12 cut-off point [11,20]. The PVR of Vis-Screen attained a sensitivity of 65% at the 6/60 cut-off point. At the same time, the other apps, such as Peek Acuity and Smart Vision Screening Instrument, reported higher sensitivity values, each scoring 85%. However, the selected age group of participants differed between the studies at a similar 6/60 cut-off point [12,21]. Compared with our app, lower sensitivity values for PVR at the 6/60 cut-off point were mainly due to the smaller number of participants with severe impairments. Even so, the moderate sensitivity of Vis-Screen was still considered satisfactory. 

Unlike the sensitivity values, the specificities achieved by PVR were consistently high at all the cut-off points. Compared with Peek Acuity at the 6/12 cut-off point, the specificity of PVR was slightly higher with 94%, while Peek Acuity reported sensitivities between 83% and 91% [11,20]. At the 6/60 cut-off point, the specificity of PVR was 98%, while specificities of 85% and 92% were reported for Peek Acuity and Smart Vision Screening Instrument, respectively [12,21]. Higher specificity values of PVR were influenced mainly by the more significant number of participants with normal vision. Nevertheless, the PVRs consistently high specificity values indicated that our app was highly specific in ruling out the normal participants, irrespective of any cut-off points. Unlike other apps that are commonly reported as single cut-off points, our findings were based on the WHO classification. Indeed, inadequate validity reports were also noticed from the others; thus, no other comparison could be made.

For conventional charts, various validity findings were observed in the literature. In Singapore, the sensitivity and specificity of the non-illuminated ETDRS chart for detecting refractive errors among school children at the 6/12 cut-off point were 70% and 96%, respectively [22]. Similar sensitivities and specificities of Tumbling E were also observed in the visual acuity screening among school-age and preschool children in India. At the 6/12 cut-off point, the reported sensitivity and specificity were 92% and 72%, respectively, for school-age children, while they were 90% and 69% for preschool children [23,24]. However, none of the previous literature mentioned the validity of these conventional charts for distance testing based on the 6/60 cut-off point. Regardless of the slight differences in the presented optotypes between conventional charts and our app, the sensitivity and specificity values attained by Vis-Screen were indeed, reasonably excellent and equivalent with both conventional and smart device-based charts.

Till the present year, no other apps have applied a pinhole measurement in their algorithm. The use of pinholes in this study was primarily to look for any improvements in the reduced acuities within the setting of vision screening. In standard practices, the pinhole was used to postulate the probability of decreased vision due to refractive errors or ocular pathologies [25]. However, we did not intend to determine our participant’s underlying causes of visual impairment. The declination of sensitivity and specificity values observed from our study was similar to a previous study by Cook et al., where the Snellen visual acuity was almost the same, either with or without the pinhole, among cataract and glaucoma patients [26]. Although the effectiveness of the pinhole test remains debatable, the globally wide use of the pinhole measurement in vision screening is still remarked as acceptable among general practitioners [26,27,28].

Generally, predictive values are frequently associated with the disease prevalence in the study population. A high PPV was often highly related to a higher disease prevalence. PVR achieved the highest PPV at the 6/12 cut-off point with the values of 82% and generally high NPVs for all cut-off points. With the use of a pinhole, the highest PPV for CVR was at the 6/12 cut-off point with 69%, and excellent NPVs were observed at all cut-off points. However, due to the difference in the disease prevalence and age of participants between the studies, no direct comparison was made among the apps. Peek Acuity reported their PPV values between 23% and 43% at the 6/12 cut-off point, while Smart Vision Screening Instrument reported 73% at the 6/60 cut-off point [11,20,21]. Smart Vision Screening Instrument reported the highest disease prevalence, 34%, while Peek Acuity reported the lowest prevalence, between 4% and 5% [20,21]. The NPVs of Peek Acuity for the 6/12 cut-off point were between 85% and 99%, and Smart Vision Screening Instrument reported 95% at the 6/60 cut-off point [11,20,21]. According to each prevalence, the predictive values obtained by both Peek Acuity and Smart Vision Screening Instrument were relatively proportionate. Even though our study involved a lower prevalence, the highest PPV values reported by Vis-Screen at the 6/12 cut-off point compared with the other two apps and the relatively high NPVs were influenced mainly by the most significant number of participants with normal vision compared with the visually impaired ones.

ROC analysis helped to improve the accuracy findings in this study. Typically, the primary role of the ROC analysis is to determine the performance of a screening or diagnostic test. The excellent AUC scores of Vis-Screen at all of the cut-off points showed that our app was good at discriminating between ‘normal’ and ‘diseased’ participants, regardless of the visual levels. No other visual acuity apps reported their findings as a ROC analysis except for Smart Vision Screening Instrument. However, we performed our analysis based on cut-off points while they based theirs on age groups [21]. The selection of 6/12 as the optimal cut-off point for Vis-Screen was only meaningful for our participants and it did not represent the whole population. The use of 6/12 as a cut-off referral was still widely practiced globally within many of the community vision screening and surveys [29,30,31].

On the other hand, reliability was more particular about the measurements’ consistency. In general, test–retest reliability was performed to determine the correlation between the measured values taken at different times. However, due to the time limitation for our screening program, the original test–retest reliability could not be performed and replaced by an ‘intra-rater’ for a similar purpose. Cohen’s kappa findings of 0.85 and 0.79 for the intra-rater of PVR and CVR showed a strong agreement of measurement taken by the same rater. Meanwhile, slightly reduced Fleiss kappa values were observed among different raters (inter-rater). For many visual acuity screening studies, the reliability or agreement regarding raters were least reported. Regarding the test–retest reliability, various statistical findings were observed in the literature. For such, Peek Acuity reported their findings as a Pearson correlation coefficient of 0.93. At the same time, Eye Chart Pro had intra-class correlations of 0.99 [12,32]. Even though there was a lack of findings from the other validated apps, the kappa scores attained by Vis-Screen for the intra-rater were still considered as good as the others. 

Unlike the test–retest reliability, there were no other reports for the inter-rater test. We could not afford to maintain the same examiners throughout all our screening programs; hence, the examiners assigned for the inter-rater part were inevitably changed from one screening to another. Therefore, the slight declination in the kappa values for the inter-rater compared with the intra-rater was within our expectation. On the other hand, only a moderate agreement was attained between Vis-Screen and the Snellen chart for the app’s reliability. As for Peek Acuity, various statistical findings between their app and the Snellen chart were observed. As such, Bastawrous et al. in 2015 reported a Pearson correlation coefficient of 0.95, Irawati et al. in 2020 reported a Cohen’s kappa value of 0.65, and Bhaskaran et al. in 2022 reported an intraclass correlation coefficient (ICC) of 0.98 [3,12,33]. Meanwhile, other apps such as Eye Chart reported an ICC between 0.74 and 0.88, Eye Chart Pro reported an ICC of 0.99, and Vision at Home as a tolerant quadratic weighted a kappa between 0.74 and 0.95 [32,34,35]. Despite all the differences, the moderate agreement of Vis-Screen was still comparable with the validated and recognized app. Above all, Vis-Screen showed a good agreement for both the raters and charts. 

Regarding the test duration, the mean time of about 70 s to complete all the Vis-Screen tests showed that the test was performed quickly and reasonably. Peek Acuity reported a mean test duration between 56 and 125 s, whereby a more extended test duration was recorded among their pediatric participants compared with older adults [11,12,36]. For conventional chart such as ETDRS, the average testing time among individuals with normal vision was about 53 s, while a more extended time was observed among individuals with ocular diseases [37]. An almost similar time range was also obtained for the Snellen chart, which was between 47 and 110 s [38,39]. Therefore, the mean test time for Vis-Screen was considered acceptable and suitable for vision screening purposes. 

### Limitation

We did not specify any age groups or ocular diseases to be included in our study participants. Even though the use of minimum cut-off point 6/12, especially among school children, may raise debates, all cut-offs used for the built-in algorithm of Vis-Screen were based on the latest recommendation by the WHO to represent the visual impairment and blindness in the general population that involved all age groups. 

## 5. Conclusions

Vis-Screen app has demonstrated itself to be valid and reliable, comparable with the Snellen chart in detecting individuals with visual impairment and blindness, and suitable for use in terms of for vision screening purposes. 

## Figures and Tables

**Table 1:** The WHO and Vis-Screen definition based on the 11th (ICD) International Classification of Visual Impairment and Blindness, and cut-off points used in the study.

Category	Definition		Cut-Off Point	Diagnostic Term Applied	
	WHO Criteria	Vis-Screen Criteria		No Disease	Disease
Normal	Distance VA 6/12 or better	Can see 6/12	NA	NA	NA
Mild VI	Distance VA worse than 6/12 but equal to or better than 6/18	Can see 6/18 but cannot see 6/12	6/12	Normal	Mild VI or worse
Moderate VI	Distance VA worse than 6/18 but equal to or better than 6/60	Can see 6/60 but cannot see 6/18	6/18	Mild VI or better	Moderate VI or worse
Severe VI	Distance VA worse than 6/60 but equal to or better than 3/60	Can see 3/60 but cannot see 6/60	6/60	Moderate VI or better	Severe VI or worse
Blindness	Distance VA worse than 3/60 up till no light perception	Cannot see 3/60	3/60	Severe VI or better	Blindness

VI: visual impairment, VA: visual acuity, and NA: not applicable.

**Table 2:** Summary of the sensitivity, specificity, predictive values, and accuracy for the Vis-Screen app against the gold standard, the Snellen chart (*n* = 408).

Cut-Off Point		Sensitivity (95% CI)	Specificity (95% CI)	PPV (95% CI)	NPV (95% CI)	Accuracy (95% CI)
6/12	PVR	88.4% (85.3, 91.5)	94.7% (92.6, 96.9)	81.7% (78.0, 85.5)	96.8% (95.1, 98.5)	93.4% (90.5, 95.6)
	CVR	85.4% (81.9, 88.8)	95.6% (93.7, 97.6)	68.6% (64.1, 73.1)	98.3% (97.1, 99.6)	94.6% (91.9, 96.6)
6/18	PVR	82.0% (78.2, 85.7)	95.7% (93.7, 97.7)	76.9% (72.8, 81.0)	96.8% (95.1, 98.5)	93.6% (90.8, 95.8)
	CVR	73.9% (69.7, 78.2)	96.9% (95.2, 98.6)	58.6% (53.8, 63.4)	98.4% (97.2, 99.6)	95.6% (93.1, 97.4)
6/60	PVR	64.7% (60.1, 69.3)	98.0% (96.6, 99.3)	57.9% (53.1, 62.7)	98.5% (97.3, 99.7)	96.6% (94.3, 98.1)
	CVR	60.0% (55.3, 64.8)	98.8% (97.7, 99.8)	37.5% (32.8, 42.2)	99.5% (98.8, 100.0)	98.3% (96.5, 99.3)
3/60	PVR	55.6% (50.7, 60.4)	99.3% (98.4, 100.0)	62.5% (57.8, 67.2)	99.0% (98.0, 100.0)	98.3% (96.5, 99.3)
	CVR	66.7% (62.1, 71.2)	99.0% (98.1, 100.0)	33.3% (28.8, 37.9)	99.8% (99.3, 100.0)	98.8% (97.2, 99.6)

VI: visual impairment, PVR: presenting vision of the right eye, CVR: corrected vision of the right eye, PPV: positive predictive value, NPV: negative predictive value, CI: confidence interval, α was set at 0.05.

**Table 3:** Summary of ROC analysis for the Vis-Screen app (*n* = 408).

Cut-Off Point		AUC (95% CI)	Correctly Classified (%)	LR+	LR−
6/12	PVR	0.93 (0.89, 0.97)	93.4	16.73	0.12
	CVR	0.91 (0.86, 0.97)	94.6	19.58	0.15
6/18	PVR	0.95 (0.92, 0.98)	93.6	18.96	0.19
	CVR	0.98 (0.96, 0.99)	95.6	23.71	0.27
6/60	PVR	0.96 (0.94, 0.99)	96.6	31.63	0.36
	CVR	0.97 (0.94, 1.00)	98.3	48.36	0.41
3/60	PVR	0.97 (0.95, 1.00)	98.3	73.89	0.45
	CVR	0.98 (0.96, 1.00)	98.8	67.50	0.34

AUC: area under the curve, CI: confidence interval, LR+: positive likelihood ratio, LR−: negative likelihood ratio, VI: visual impairment, and null hypothesis: true area = 0.5.

**Table 4:** Summary results of intra-user, inter-user, and app’s reliability for the Vis-Screen app (*n* = 408).

Category	Vision Test	Kappa Value (95% CI)	Z	*p*-Value
Intra-user	PVR	0.85 (0.80, 0.91)	24.77	˂0.001
	CVR	0.79 (0.70, 0.89)	22.28	˂0.001
Inter-user	PVR	0.72 (0.66, 0.77)	36.31	˂0.001
	CVR	0.67 (0.60, 0.75)	32.79	˂0.001
Vis-Screen vs. Snellen chart	PVR	0.61 (0.53, 0.68)	17.96	˂0.001
	CVR	0.52 (0.42, 0.62)	14.89	˂0.001

CI: confidence interval.

## Data Availability

The corresponding author will provide the dataset of this study upon request.
